# *Plasmodium falciparum* FIKK Kinase Members Target Distinct Components of the Erythrocyte Membrane

**DOI:** 10.1371/journal.pone.0011747

**Published:** 2010-07-23

**Authors:** Marta C. Nunes, Mami Okada, Christine Scheidig-Benatar, Brian M. Cooke, Artur Scherf

**Affiliations:** 1 Unité de Biologie des Interactions Hôte-Parasite, CNRS UR2581-Institut Pasteur, Paris, France; 2 Department of Microbiology, Monash University, Victoria, Australia; University of Minnesota, United States of America

## Abstract

**Background:**

Modulation of infected host cells by intracellular pathogens is a prerequisite for successful establishment of infection. In the human malaria parasite *Plasmodium falciparum*, potential candidates for erythrocyte remodelling include the *apicomplexan*-specific FIKK kinase family (20 members), several of which have been demonstrated to be transported into the erythrocyte cytoplasm via Maurer's clefts.

**Methodology:**

In the current work, we have knocked out two members of this gene family (*Pf fikk7.1* and *Pf fikk12*), whose products are localized at the inner face of the erythrocyte membrane. Both mutant parasite lines were viable and erythrocytes infected with these parasites showed no detectable alteration in their ability to adhere *in vitro* to endothelial receptors such as chondroitin sulfate A and CD36. However, we observed sizeable decreases in the rigidity of infected erythrocytes in both knockout lines. Mutant parasites were further analyzed using a phospho-proteomic approach, which revealed distinct phosphorylation profiles in ghost preparations of infected erythrocytes. Knockout parasites showed a significant reduction in the level of phosphorylation of a protein of approximately 80 kDa for FIKK12-KO in trophozoite stage and a large protein of about 300 kDa for FIKK7.1-KO in schizont stage.

**Conclusions:**

Our results suggest that FIKK members phosphorylate different membrane skeleton proteins of the infected erythrocyte in a stage-specific manner, inducing alterations in the mechanical properties of the parasite-infected red blood cell. This suggests that these host cell modifications may contribute to the parasites' survival in the circulation of the human host.

## Introduction

*Plasmodium falciparum* is the species responsible for the vast majority of malaria-related morbidity and mortality. Serious clinical complications frequently arise due to dramatic modification of the structural and functional properties of *P. falciparum*-infected erythrocytes (IEs) [1]. During the intraerythrocytic stage, malaria parasites interact with and affect both the plasma membrane and the membrane skeleton of the IEs [2], [3]. Alterations in biochemical, structural and adhesive properties of the host membrane occur as the parasite develops within the erythrocyte [1], [3], [4]. Recently, a large study showed that many parasite proteins contribute at different degrees to remodel the host erythrocyte [5]. Among parasite-secreted proteins, several types of enzymes (like kinases, several still uncharacterized and phosphatases) are trafficked to the erythrocyte membrane indicating that post-translational modifications may contribute to establish successful intracellular parasite proliferation [6]. Phosphorylation of membrane skeleton proteins of both parasite and host origin have been described during *P. falciparum* infections [7], [8]. Selective phosphorylation of host membrane skeleton proteins include protein 4.1, β-spectrin, ankyrin and band 3 [7], [9]–[11]. It has also been established that phosphorylation of some of these proteins modulate their interactions with other membrane proteins [12], [13] and, consequently, the membrane mechanical functions and membrane stability [14], [15]. In addition, erythrocyte membrane skeleton phosphorylation was suggested to be involved in the regulation of malaria parasite invasion and development [11], [16]. However, the molecular events involved in the phosphorylation of membrane skeleton proteins have not yet been identified.

Recently we showed that some members of the *apicomplexa*-specific FIKK kinase family are transported to the erythrocyte membrane via Maurer's clefts [17]. *Fikk* is a single copy gene in most *Plasmodium* species but has expanded in *P. falciparum* to 20 related members dispersed mostly on subtelomeric regions of 11 of the 14 parasites chromosomes [18], [19]. Nineteen *Pf fikk* genes possess the *Plasmodium* exported element/host targeting motif downstream of a signal or anchor sequence required for transport across the parasitophorous vacuole [20], [21]. Despite the fact that these proteins share a common structure, the N-terminal regions are highly variable, suggesting that individual members of this family may have access to distinct substrate pools since their variable N- terminal can probably target them to different locations. Due to the restricted homology with well-characterized kinase domains, the FIKK proteins did not cluster within any of the kinase groups described in higher eukaryotes [19].

In this work, we have analyzed the biological role of two members of the *P. falciparum* FIKK kinase family (FIKK7.1 and FIKK12) in IEs. We show that both FIKK kinases are non-essential for parasite growth *in vitro*. However, the absence of functional copies of either *Pf fikk7.1*(*MAL7P1.144*) or *Pf fikk12* (*PFL0040c*) resulted in altered rigidity of the IEs using single cell micromanipulation and both mutant parasites showed changes in the phosphorylation pattern of two distinct proteins of the erythrocyte membrane skeleton.

## Results

### Single targeted disruption of two *fikk* genes in *P. falciparum* leads to viable blood stage development

**Figure 1:**
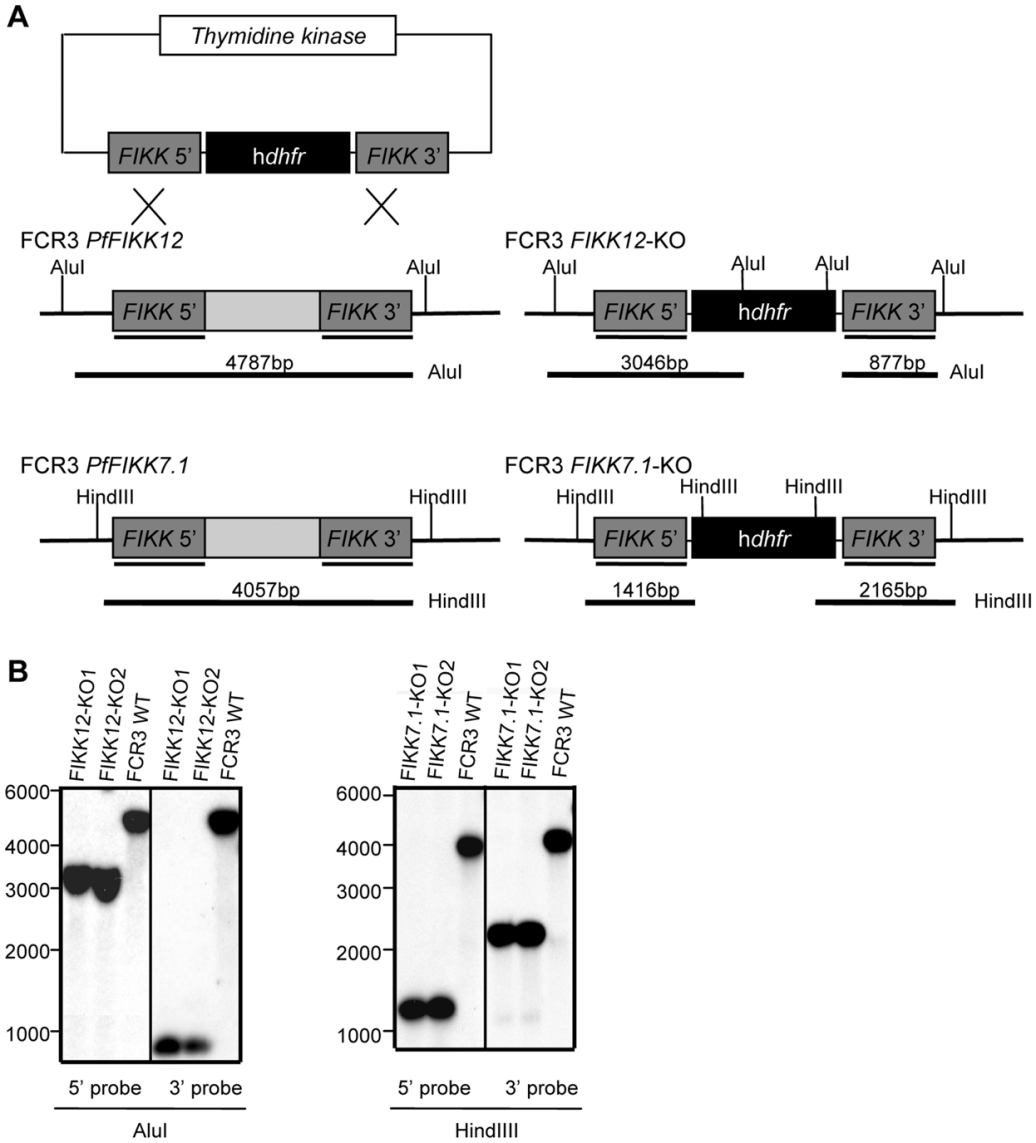
The *Pf fikk7.1* and *Pf fikk12* genes were disrupted by a double-crossover event. A. Schematic representation of the *Pf fikk* loci in the FCR3 wild type and FIKK-KO parasites. The plasmid constructs, pHTK-FIKK, contain two portions of the specific *Pf fikk* gene (dark grey portions), which were cloned in the same direction flanking the *hdhfr* cassette. The crosses indicate the two regions where homologous integration has occurred, here represented for *Pf fikk12*. The restriction sites are shown both for the wild type gene and the mutant gene. B. Southern blot analysis of the parental FCR3 strain and two representative clones of both FIKK12-KO (left panel) and FIKK7.1-KO (right panel). Hybridizations were carried out with 5' and 3' probes. The position of the probes is shown in A.

We have previously reported that FIKK proteins are exported to different locations in the IE. Using both GFP-tagging and specific antibodies against FIKK12 we noticed that this protein was transported to the erythrocyte membrane [17]. In this initial analysis we also noticed that *Pf fikk7.1* was more than 3-fold up-regulated in ring stage FCR3-CSA-selected (chondroitin sulfate A) parasites when compared to CD36-selected parasites. To investigate the biological role of these two members of the *Pf fikk* gene family, we established two parasite lines with single gene disruption by double crossover recombination. The pHTK-FIKK7.1 and the pHTK-FIKK12 vectors [22] contain the human dihydrofolate reductase (*hdhfr*) gene flanked by 5′ and 3′ segments of the *Pf fikk7.1* and *Pf fikk12* genes, respectively (Fig. 1A). FCR3 parasites were transfected with the pHTK-FIKK constructs and selected on WR99210 and ganciclovir to generate two insertional disruptant mutants, the FIKK7.1-KO and the FIKK12-KO. After drug selection, the mutants were cloned by limiting dilution and genetically characterized. Clones were screened by polymerase chain reaction (PCR) analysis for the disruption of the *Pf fikk7.1* or *Pf fikk12* gene as well as for the absence of contaminating wild type gene (data not shown). To confirm that the pHTK-FIKK vector had integrated into the respective *Pf fikk* gene, Southern blots were performed using genomic DNA derived from parental FCR3 or recombinant parasites previously digested with AluI to test FIKK12-KO or HindIII to test FIKK7.1-KO. Radiolabelled probes from *Pf fikk7.1* 5′ and 3′ segment or *Pf fikk12* 5′ and 3′ segment were used for hybridization. These hybridizations showed bands of the expected size, indicating that the integration occurred at the predicted site within the *Pf fikk* genes (Fig. 1B). Transcription of the specific genes was analysed by real-time PCR. No transcript was detected in the respective KO-line; i.e. no *Pf fikk7.1* transcript was detected in the FIKK7.1-KO line and no *Pf fikk12* transcript was detected in the FIKK12-KO line.

The KO parasites were viable and there was no obvious difference in the parasite growth and multiplication rates between the KO parasites and wild type FCR3 strain (data not shown), suggesting the FIKK7.1 and FIKK12 proteins are non-essential for the parasite growth of laboratory isolates.

### Analysis of the adhesive properties of IEs and trafficking process in FIKK-KO parasites

**Figure 2:**
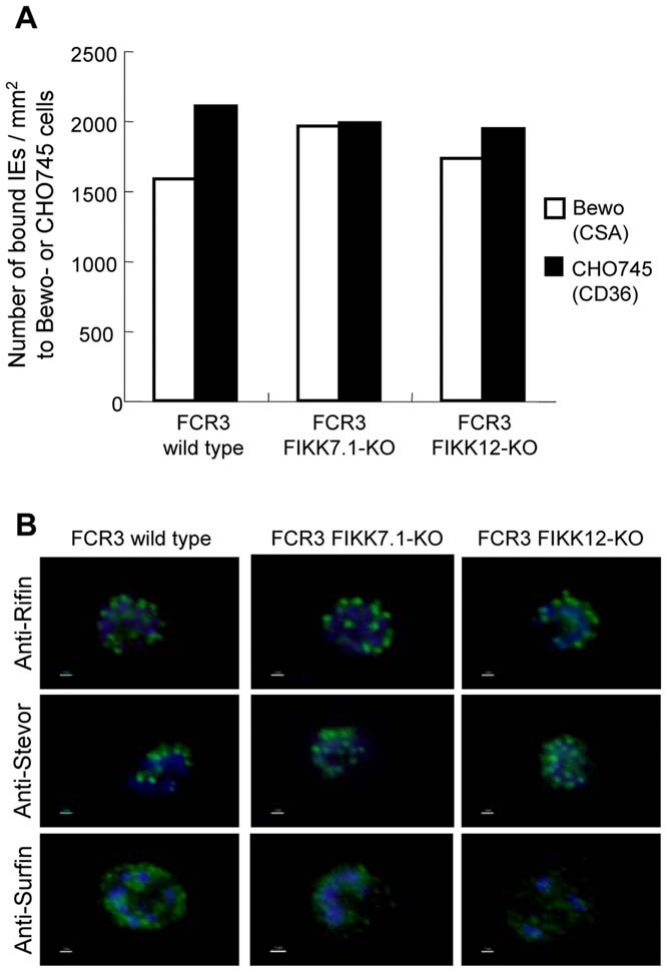
Characterization of the FIKK-KO parasites. A. The adhesion phenotypes of the two KO parasite lines were investigated. The FIKK7.1- and FIKK12-KO parasites and wild type FCR3 were selected on cells expressing either CSA (white bar) or CD36 (black bar), after 3 rounds of selection all the three parasite lines showed similar binding densities on BeWo (CSA) or CHO745-CD36 cells. B. Cellular localization of A-type Rifin, Stevor and Surfin 4.2 in FIKK-KO and wild type parasites. Antigen was detected using specific anti-Rifin, Surfin or Stevor antibodies by immunofluorescence assay.

Given the transit of several FIKK kinases via Maurer's clefts and the presence of a variable N-terminal domain in each protein we hypothesized that the kinase activity of these proteins might be involved in the trafficking of parasite-encoded variant surface molecules, such as the adhesion molecule *Plasmodium falciparum* erythrocyte membrane protein 1 (PfEMP1). To test this hypothesis we analyzed the adhesive properties of the transgenic lines to endothelial cell-expressed receptors by static *in vitro* adhesion assays. The FIKK7.1-KO and FIKK12-KO parasite lines were selected to adhere to human placenta choriocarcinoma Bewo cells expressing CSA receptor or Chinese hamster ovary 745 cell expressing CD36 receptor [23]. The levels of parasite cytoadherence were quantified after the third round of selection (Fig. 2A). Both KO parasite lines cytoadhered to CSA and CD36 in similar numbers to the wild type parasites, suggesting that the disruption of these *Pf fikk* genes neither prevented the trafficking of PfEMP1 to the surface of the infected erythrocyte membrane nor their ability to bind to different receptors.

To examine if the PfEMP1 switching rates in the KO parasites were altered, we performed binding assays with receptors immobilized on plastic over a 2-month period after which the numbers of bound IEs per mm^2^ were quantified. The binding phenotype of the CSA-selected KO parasites was analyzed with two different receptors (purified bovine CSA and human recombinant CD36) at three different intervals: 1 week, 5 weeks and 7 weeks post-selection (Supplementary Figure S1). Both KOs and wild type parasites maintained similar CSA-binding phenotypes during the long *in vitro* 7 weeks cultivation and they did not bind to CD36, though as expected the binding efficiency to CSA decreased gradually in the three CSA-selected parasite lines. CD36-selected parasites conserved a strong affinity for CD36 during the all experiment (data not shown). These data suggest that FIKK12 and FIKK7.1 do not interfere with parasite adhesion to CSA and CD36 receptors. Furthermore, the assay we used did not reveal any apparent alteration in the switching rate to other adhesion phenotypes.

We also tested the possibility that the FIKK7.1 and/or FIKK12 might be involved in the trafficking of other parasite-encoded variant multi-gene families. To address this question, the localization of Rifin (repetitive interspersed family) [24], Stevor (subtelomeric variable open reading frame) [25] and Surfin (surface-associated interspersed family) [26] proteins was analyzed in the transgenic parasites and compared to wild type parasites by immunofluorescence assay using specific antibodies (see Materials and Methods). The genes of these three families are located close or within the subtelomeric chromosomic region. And different protein members have been localized in the IEs Maurer's clefts. Similar immunofluorescence patterns were detected in IEs with mature stages of FIKK7.1-KO, FIKK12-KO and FCR3 parental parasites (Fig. 2B). We were unable to detect differences in the Maurer's cleft localizations of A-type Rifin, Surfin 4.2 and Stevor between the KO and wild type lines, suggesting that FIKK7.1 and FIKK12 do not control trafficking of these multigene families into the erythrocyte membrane.

### Erythrocytes infected with mutant FIKK7.1-KO and FIKK12-KO parasites have altered membrane rigidity

**Figure 3:**
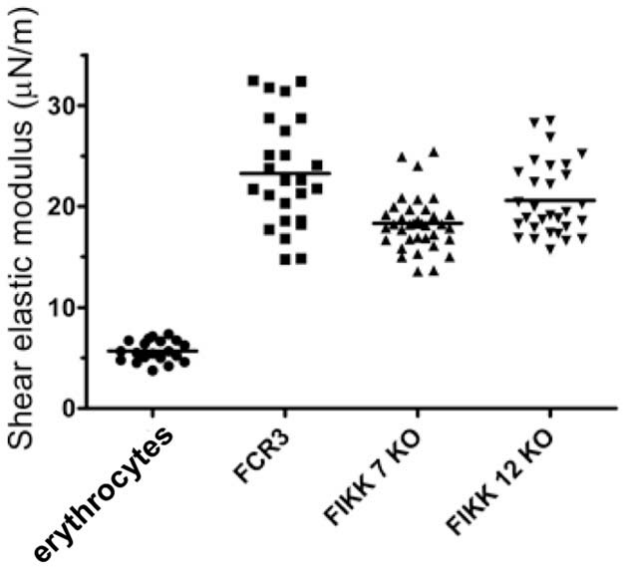
Effect of FIKK7.1 and FIKK12 on the membrane shear elastic modulus of *P. falciparum* IEs. Membrane shear elastic modulus of individual uninfected erythrocytes or IEs (FCR3, FIKK7.1-KO or FIKK12-KO) was measured by micropipette aspiration. Uninfected erythrocytes are non-parasitized erythrocytes taken from parasite cultures. Each point represents the shear elastic modulus for an individual erythrocyte. Solid horizontal bars represent the mean of all data in each group.

To evaluate whether FIKK proteins play a role in alteration of the mechanical properties of the IE membrane skeleton and, therefore overall erythrocyte deformability, we measured and compared the shear elastic modulus of uninfected erythrocytes and highly synchronized erythrocytes infected with pigmented trophozoites of either parental FCR3 parasites or mutant parasite lines (Fig. 3). Using single cell micromanipulation, we observed that infection of erythrocytes with *P. falciparum* FCR3 caused an approximately 4-fold increase in the rigidity of the erythrocyte membrane. In contrast, the overall level of rigidification of erythrocytes infected with either of the FIKK-KO parasites was reduced when compared to erythrocytes infected with parental FCR3 parasites. When compared to the level of rigidification of the erythrocyte membrane induced by FCR3 parental parasites, the rigidity of erythrocytes infected with FIKK7.1-KO parasites was significantly lower (P<0.0005 by Mann Whitney U test). Similarly, the median level of rigidity of erythrocytes infected with FIKK12-KO parasites was also lower than those infected with FCR3 but the difference did not quite reach statistical significance at the 95% confidence level (P = 0.06) These suggest that both FIKK7.1 and FIKK12 play a role in modulating the overall level of membrane rigidification induced by *P. falciparum* infection.

### Phosphorylation levels of two different proteins in the erythrocyte membrane skeleton are altered in FIKK12-KO and FIKK7.1-KO parasites

We took a phospho-proteomics approach in an attempt to identify proteins which are regulated by the FIKK kinases. Since most of the FIKK members are transported into the erythrocyte membrane via Maurer's clefts, we investigated the level of phosphorylation in ghost fractions prepared from IEs. We first evaluated the purity of the ghost samples, confirming that only erythrocyte membranes and Maurer's cleft were present without contamination by other parasite components. The punctate pattern of Maurer's cleft proteins revealed by antibodies against the Pf332 antigen was observed in the ghost preparations as previously described [27] and the intraparasitic maker Pfhsp70 was detected in the total IEs extracts but not in the ghost fraction, indicating no contamination of parasite cytosolic proteins in the ghosts (Supplementary Figure S2).

**Figure 4:**
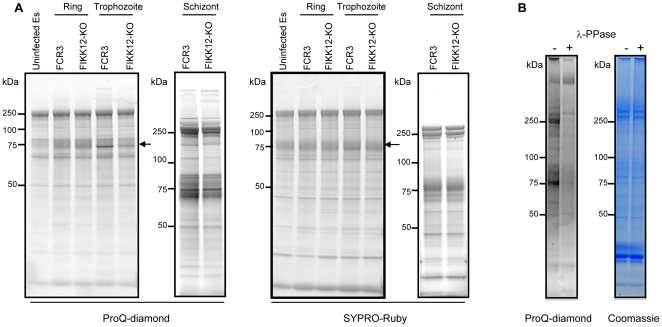
FIKK12-KO changes the phosphorylation pattern of a 80 kDa protein. A. Phosphorylation pattern in the ghost fraction of FIKK12-KO parasite. Ghost fractions from uninfected erythrocytes and ring, trophozoite and schizont stages of FCR3 and FIKK12-KO IEs were subjected to SDS-PAGE. The gel was stained with ProQ-diamond (left panel) and then stained with SYPRO-Ruby (right panel). Arrow indicates a band of 80 kDa protein. B. Control of the specificity of ProQ-diamond. The ghost fraction from trophozoite stage of FCR3 was incubated with lambda protein phosphatase, subjected to SDS-PAGE, and stained with ProQ-diamond. The gel was then stained with Coomassie blue.

The purified ghost fractions from ring (10–14 hours after infection), trophozoite (18–22 hours after infection) and schizont (32–36 hours after infection) stages of the FIKK12-KO and the parental FCR3 parasites were analyzed by gel-based 1 dimensional electrophoresis (1-D). Given that phosphorylation in *P. falciparum* blood stages occurs in a stage-specific manner [8], [10] tightly synchronized parasites, within a window of +/− 2 hours, were used in this analysis. FIKK12-KO and FCR3 ghosts from the three intraerythrocytic stages were lysed in the presence of protease and phosphatase inhibitors, after which the same protein amount was subjected to 1-D SDS-PAGE. The gel was sequentially stained with the phosphoprotein-specific Pro-Q Diamond fluorescence dye [28], [29] followed by SYPRO Ruby staining to detect total protein. The experiment was performed independently three or four times in each stage. Comparison of the phosphorylation pattern between the KO and wild type parasites preparations showed a clear distinct profile. A protein with an apparent molecular weight of 80 kDa was significantly less stained in FIKK12-KO trophozoites than in wild type parasites (Fig. 4A). This differential phosphorylation level was reproducibly observed at the trophozoite stage but neither in ring nor schizont stages.

To investigate if the detected change in phosphorylation could result from non-specific protein staining by Pro-Q Diamond, the FCR3 ghost sample was treated with λ–protein phosphatase (λ-PPase), a Mn^2+^-dependent enzyme with activity towards phosphorylated serine, threonine, tyrosine, and histidine [30]. Treated and untreated ghost samples were resolved by 1-D and compared by Pro-Q Diamond and Coomassie blue staining (Fig. 4B). With the exception of one protein with high molecular weight (over 250 kDa), all proteins were sensitive to λ-PPase treatment and showed a strong reduction, or no reactivity to the phospho-specific Pro-Q Diamond staining, validating the specificity of the Pro-Q Diamond staining.

**Figure 5:**
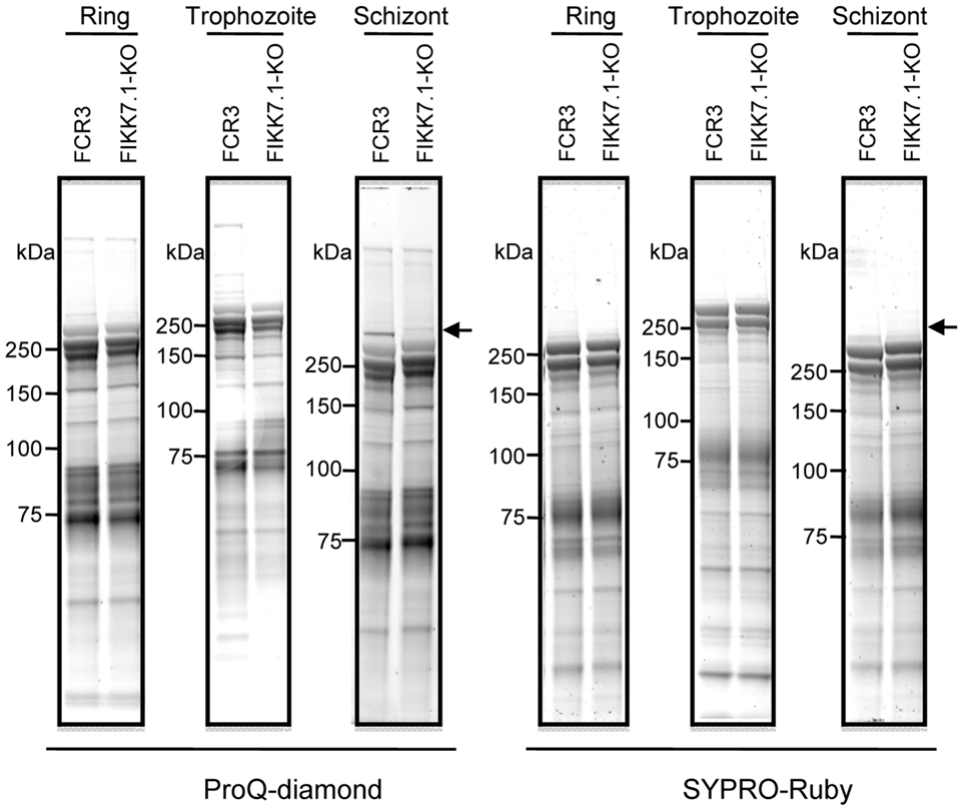
FIKK7.1-KO changes phosphorylation pattern in a 300 kDa protein. The ghost fractions from ring, trophozoite and schizont stages of FCR3 and FIKK7.1-KO IEs were subjected to SDS-PAGE and stained with ProQ-diamond (left panel) and then stained with SYPRO-Ruby (right panel). Arrow indicates a band of 300 kDa protein.

FIKK7.1-KO ghost extracts from highly synchronized parasites were also analyzed for changes in the phosphorylation pattern. We detected a band with an estimated molecular weight of 300 kDa in schizont extracts from wild type ghosts, which was absent in FIKK7.1-KO extracts (Fig. 5). No difference was seen in the 80 kDa band, indicating that FIKK7.1 and FIKK12 target distinct proteins in the erythrocyte membrane skeleton. This differential phosphorylation level was reproducibly observed at the schizont stage but neither in ring nor trophozoite stages.

## Discussion

In this work, we demonstrate that two members of the FIKK kinase family are involved in the remodeling of erythrocyte membrane skeleton proteins. Importantly, each analyzed FIKK member apparently targets a distinct protein at a different time point of the asexual blood cycle. Our experimental evidence suggests that FIKK12 targets a protein of 80 kDa at trophozoite stage and FIKK7.1 targets another protein of approx. 300 kDa at schizont stage. A recent study reported changes in the phosphoproteome of IEs and identified numerous *P. falciparum* phosphorylated proteins and 77 human proteins as phosphorylated in IEs whereas only 48 were detected in uninfected erythrocytes [8]. This indicates an elevated level of post-translational modifications of the host cell by parasite kinases. Given that most of the *Pf fikk* genes were shown to be transcribed in blood stages [17] and N-terminal regions are unique to each paralog, this raises the possibility that each FIKK protein might have different functions in the IEs and that other FIKK proteins could have other biological roles including trafficking, adhesion and antigenic variation.

A reverse genetic screen identified different parasite proteins that are related with changes in the deformability of the erythrocyte membrane [5]. Inactivation of several genes (none of the *fikk* genes was analyzed) showed moderate decreases in the deformability of the IEs, validating that several proteins contribute to the overall IEs rigidity. We may expect that these proteins interact with the erythrocyte membrane skeleton or may facilitate the trafficking of skeleton-related proteins. We previously showed that FIKK12 is exported into the cytoplasm of the erythrocyte via Maurer's cleft at ring stage and disassociates from the Maurer's cleft at trophozoite stage [17]. Taken together, this raises the possibility that the exported FIKK12 protein might interact with the membrane skeleton of IEs at the trophozoite stage and be involved in the phosphorylation of proteins of the erythrocyte membrane skeleton. Potential candidates for FIKK12 substrates include the erythrocyte protein protein 4.1 with a molecular weight of 80 kDa. Protein 4.1 plays different roles in protein attachment to the membrane and is involved in regulating the membrane mechanical stability, which is decreased in the protein 4.1-deficient erythrocytes [31], [32]. Interestingly phosphorylation of protein 4.1 has been suggested to affect its role in protein-protein interaction at the erythrocyte membrane skeleton and erythrocyte membrane deformability [33]. Our results show that disruption of *Pf fikk12* resulted in less phosphorylation of the band at 80 kDa, however this was not completely blocked. One possibility is that this substrate might be a substrate for a variety of kinases in the erythrocyte and that FIKK12 might phosphorylate a particular site.

For FIKK7.1 we identified a potential substrate with a high molecular weight, although the parasite protein PfEMP1 has a molecular weight in this range and is only detectable after radioactive labelling, at this stage we cannot conclude that a parasite protein in the erythrocyte membrane is the target of FIKK7.1. Furthermore, we do not know if these FIKK kinases target directly erythrocyte membrane proteins or are involved in a phosphorylation cascade. At this stage, we can not rule out the possibility that differences in the phosphoproteomes of the KO lines are derived from the lack of activity of non FIKK kinase regulated by FIKK12 or 7.1.

Deletion of both *Pf fikk7.1* and *Pf fikk12* in the FCR3 strain demonstrates that they are not crucial for parasite replication *in vitro*, and since the transgenic parasite lines show normal multiplication rates they are probably not involved in the invasion process. However, our erythrocyte deformability data strongly suggest that these proteins are involved in changes of the erythrocyte membrane skeleton to meet the needs of the growing intracellular parasite. The resulting modification of the rheological properties of IEs and may have a role in splenic clearance by IEs *in vivo*.

When we commenced this work, we hypothesized that the FIKK proteins could be involved in the process of IE adhesion and antigenic variation of *var* genes, since this process is unique to *P. falciparum*. It was recently reported that the phosphorylation of PfEMP1 cytoplasmic domain by casein kinase II alters the association of this domain with knob associated histidine-rich protein and interestingly that inhibition of phosphorylation reduced the cytoadherence of IEs to two endothelial receptors [34]. We, however, could not see any differences in the adhesion properties, switching rate and trafficking of multigene families between the two KO parasite lines and the wild type FCR3 strain.

In conclusion, we present for the first time that FIKK kinases are likely involved in remodelling the membrane of IEs and our data point to changes in the cellular mechanical properties in a stage-specific and target specific manner. Work is ongoing to identify the specific proteins that are phosphorylated by these FIKK kinases. In the future it will be interesting to investigate if the *Pf fikk* genes perform similar functional roles in gametocytes and infected hepatocytes.

## Materials and Methods

### *Plasmodium falciparum* cultures and adhesion assays

*P. falciparum* blood stage parasites from the FCR3 strains were cultured using modifications to the method described by Trager and Jensen [35]. IEs were selected on the human placental derived BeWo cell line (European Collection of Cell Cultures) to obtain the FCR3-CSA parasite line [23] or on Chinese Hamster Ovary cells-745 (CHO-745; American Type Culture Collection) expressing CD36 [36] to obtain the FCR3-CD36 parasite line. Pannings were repeated three times, and parasites were tested for their ability to bind purified CSA (Sigma) or recombinant human CD36 (R&D Systems). Cytoadhesion assays on receptors immobilized on plastic petri dishes were carried out as described [37], [38]. Briefly, plastic Petri dishes were coated overnight at 4°C with phosphate-buffered saline (PBS) containing 1 mg/ml CSA sodium salt from bovine trachea (Sigma), 1 mg/ml chondroitin sulfate C sodium salt from shark cartilage (Sigma), 10 µg/ml recombinant human CD36 (R&D Systems). All spots were blocked with 1% bovine serum albumin (BSA) for 1 h at room temperature (RT) before trophozoite-IE (5×10^7^ IEs/ml) were allowed to adhere. The average number of adherent IE (±SEM) for four different fields in duplicate spots was determined in two to three independent experiments after fixing with 2% glutaraldehyde in PBS for 2 h at RT and staining the plates with Giemsa. Pictures were taken with a Nikon camera. Lucia software was used to count the number of bound IEs per mm^2^.

### Plasmids and transfection

*Pf fikk7.1* and *Pf fikk12* were targeted using fragments amplified by PCR from FCR3 strain genomic DNA with the following oligonucleotides: *fikk7.1* 5' segment 5′-GAG*ccgcgg*TAGTACATTGTATAATAAAATATAACGC-3′ and 5′-CGC*agatct*CAAGAGATTATCATTTTTATTTTGC-3′ and 3′ segment 5′-ACGC*ccatgg*CTGTGGATATGTTGTAATGGTATC-3′and 5′-CGA*cctagg*CTATAAATATAATATTATGTATGCAC-3′; *fikk12* 5′ segment 5′-GAG*ccgcgg*ATGTATATTTTGAGAAATATGTTCTG-3′ and 5′-CGC*actagt*TCGTCCTCTTTTAAATTAGACATAC-3′ and 3′ segment 5′-ACGC*ccatgg*CAGATAAATTAAGACATATAGATAAAAAG-3′ and 5′-CGA*cctagg*TTATGTTTCGTTAAACCATGGGTGTG-3′ (enzyme restriction sites in italics). These PCR fragments were sequentially cloned into pHTK [22] using the SacII/BglII and NocI/AvrII sites for *fikk7.1* and SacII/SepI and NocI/AvrII sites for *fikk12*, to derive pHTK-*FIKK7.1* and pHTK-*FIKK12*, respectively. Ring-stage FCR3-CSA *P. falciparum* parasites were transfected with 100 µg plasmid DNA and cultured with WR99210 (10 nM) (Jacobus Pharmaceutical Co. Inc.) after cultures were established parasites where double crossover homologous recombination events had occurred were selected with 4 µM ganciclovir (Roche). The resistant parasites were cloned by limiting dilution.

### Southern blot analysis of the two KO lines

Genomic DNA was digested using the following enzymes: HindIII (FIKK7.1 transfectants) and AluI (FIKK12 transfectants) and size fractionated on 0.8% agarose gels. Southern blot were performed, as described previously [39]. Specific probes were amplified with the same set primers used for the initial cloning. Membranes were hybridized at high-stringency conditions at 60°C overnight and washed twice with 0.2x saline-sodium citrate (SSC) and 0.1% SDS at 60°C for 30 min.

### Immunofluorescence microscopy

Synchronized IEs were washed in PBS, cell pellets were resuspended in 10 vols of PBS and a monolayer was set onto microscope slides. Parasites were air-dried and fixed for 30 min at RT in 4% paraformaldehyde and 0.0075% glutalaldehyde. Slides were washed with PBS and incubated with the primary antibodies diluted in 0.1% BSA: rabbit anti-Rifin-A 565 antibody 1∶100 (serum raised against a peptide in the highly conserved C-terminus of RIFIN-A, recognizes this type of RIFIN specifically), rabbit anti-Stevor antibody 1∶400 (obtained from rabbits immunized with a peptide designed on the basis of conserved STEVOR regions and recognizes the subset of *stevor* genes transcribed in parasite population) and rabbit anti-Surfin 4.2 antibody 1∶800 (antibody against PFD1160w that detects specifically Surfin 4.2) for 45 min at RT [24]–[26]. After washing cells were incubated for 30 min with mouse anti-rabbit secondary antibody conjugated with fluorochrome (Invitrogen) in a 1∶500 dilution. Slides were washed thoroughly in PBS and mounted in Vectashield anti-fading with DAPI (Vector Laboratories). Images were captures using a Nikon Elipse 80i optical microscope.

### Ghost preparation

Erythrocyte ghosts were essentially prepared as previously described with some modifications [40]. Synchronized ring (10–14 hours after infection), trophozoite (18–22 hours after infection) or schizont (32–36 hours after infection)-infected erythrocytes were extensively washed in RPMI 1640 medium and lysed for 15 min at 4°C in 40 vols of hypotonic buffer [10 mM sodium-phosphate buffer, pH7.4 containing protease inhibitor cocktail tablet (Roche) and phosphatase inhibitors, 400 nM Okadaic acid (Sigma) and phosphatase inhibitor cocktail 2 (Sigma)]. The lysates were then separated by centrifugation at 15,000×g for 30 min at 4°C into a cytosolic fraction and a pellet fraction containing ghosts and free parasites. The erythrocyte ghosts were collected at the top of the free parasite pellet, washed extensively five times in the hypotonic buffer. Ghosts were also prepared from uninfected erythrocyte for a mocked control. Ghost samples from each parasite stage were prepared in three or four independent experiments and analyzed by phospho-proteomics. Twenty µg of the purified ghosts were analyzed by SDS-PAGE using 4–12% polyacrylamide gel followed by immunoblot assay using anti-Pf332 and anti-Pfhsp70 antibodies to confirm the purity of the ghost preparation. In some experiments, 20 µg of ghost proteins were subjected to dephosphorylation prior SDS-PAGE analysis. For this samples were incubated for 30 min at 25°C with 4,000 U of λ-PPase (New England BioLabs) in reaction buffer (50 mM Tris-HCl pH7.5, 0.1 mM EDTA, 5 mM dithiothreitol, 0.01% Brij 35, and 2 mM MnCl_2_).

### Staining procedures

Polyacrylamide gels were fixed in 50% methanol, 10% acetic acid, and stained using Pro-Q Diamond phosphoprotein gel stain (Molecular Probes) in accordance with the manufacturer's instructions [41]. The stained gel was visualized on a Typhoon 9400 scanner (Amersham Biosciences) using excitation at 532 nm and 560 nm longpass emission filter, normal sensitivity (600 V), 3 mm focal plane and 50 mm resolution. Total protein was restained with SYPRO Ruby total protein gel stain (Molecular Probes), and visualized on the scanner using excitation at 457 nm and 610 nm bandpass emission filter and the same settings as above. Images were acquired as gel file format, imported into ImageJ software and stored as tiff file format for subsequent analysis.

### Measurement of membrane shear elastic modulus by micropipette aspiration

Single cell micropipette aspiration was used to determine the shear elastic modulus of erythrocyte membranes as previously described [42]. Briefly, the membrane of individual erythrocytes was aspirated progressively into glass micropipettes (internal diameter 1.3 µm) over a range of increasing negative hydrostatic pressures. The shear elastic modulus of the membrane skeleton was determined by measuring the length of a membrane tongue (L) aspirated from the erythrocyte into the pipette for a range of aspiration pressures (P) and calculated from the linear regression of dL/dP. The range of aspiration pressures was 1.0 - 4.5 mmH_2_O for uninfected erythrocytes or 1.0–12.0 mmH_2_O for infected erythrocytes. All measurements were performed at RT (approximately 20–25°C).

## Supporting Information

Figure S1 Switching rate of the KO parasites during culture. CSA-selected KO and wild type parasites were cultivated for 1, 5 and 7 weeks, and their binding ability to CSA and CD36 receptors were analyzed.
(0.06 MB PDF)

Figure S2 Verification of the purity of ghost fractions. A. Immunofluorescence analysis of the intact IEs and ghost fractions of IEs. Antigen was detected with Pf332 specific antibody. B. Immunoblot analysis of uninfected erythrocytes, ghost fractions and intact IEs. Antigen was detected using specific anti-Pf332 and Pfhsp70 antibodies.
(0.02 MB PDF)
