# Supplementary figures and images for: Plasmodium falciparum FIKK Kinase Members Target Distinct Components of the Erythrocyte Membrane
Source: PLoS One. 2010 Jul 23;5(7):e11747. doi: 10.1371/journal.pone.0011747 (PMC2909202; doi:10.1371/journal.pone.0011747)

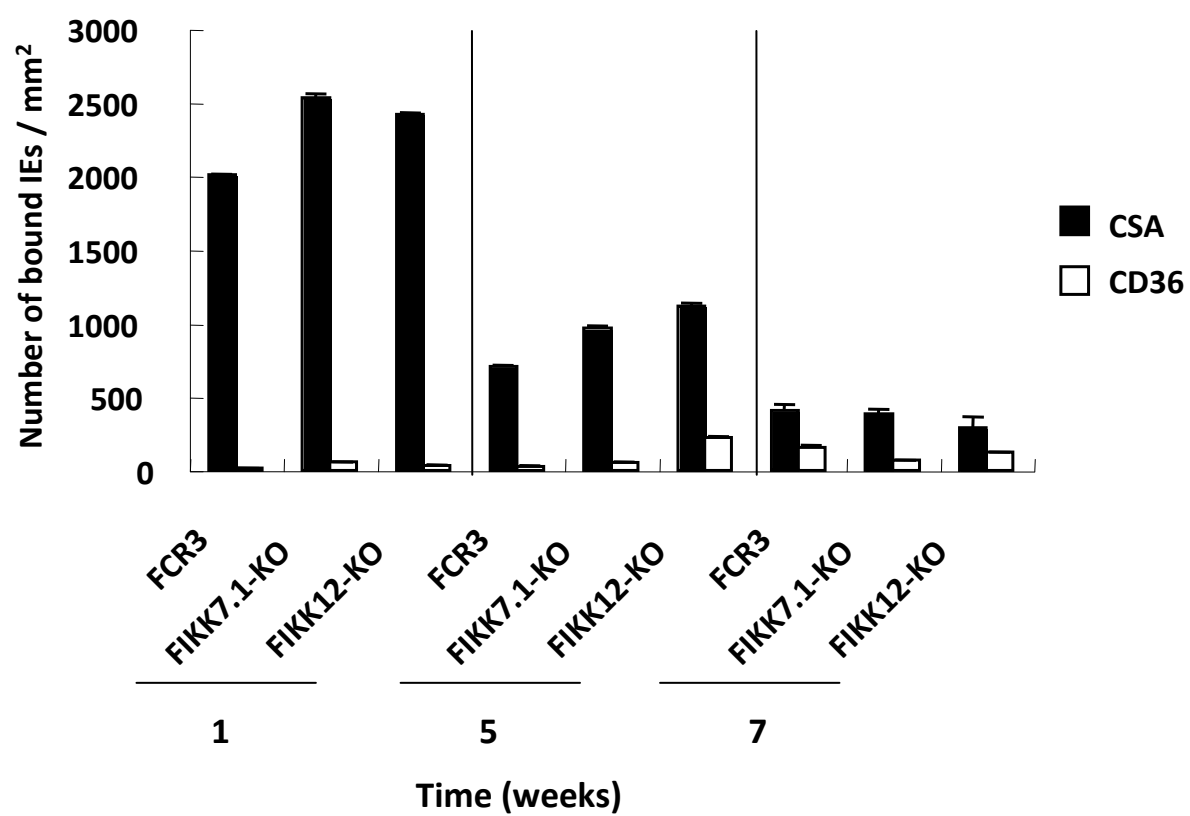

Supplementary-Figure 1

Supplement: Figure S1 — Switching rate of the KO parasites during culture. CSA-selected KO and wild type parasites were cultivated for 1, 5 and 7 weeks, and their binding ability to CSA and CD36 receptors were analyzed. (0.06 MB PDF) [file pone.0011747.s001.pdf]

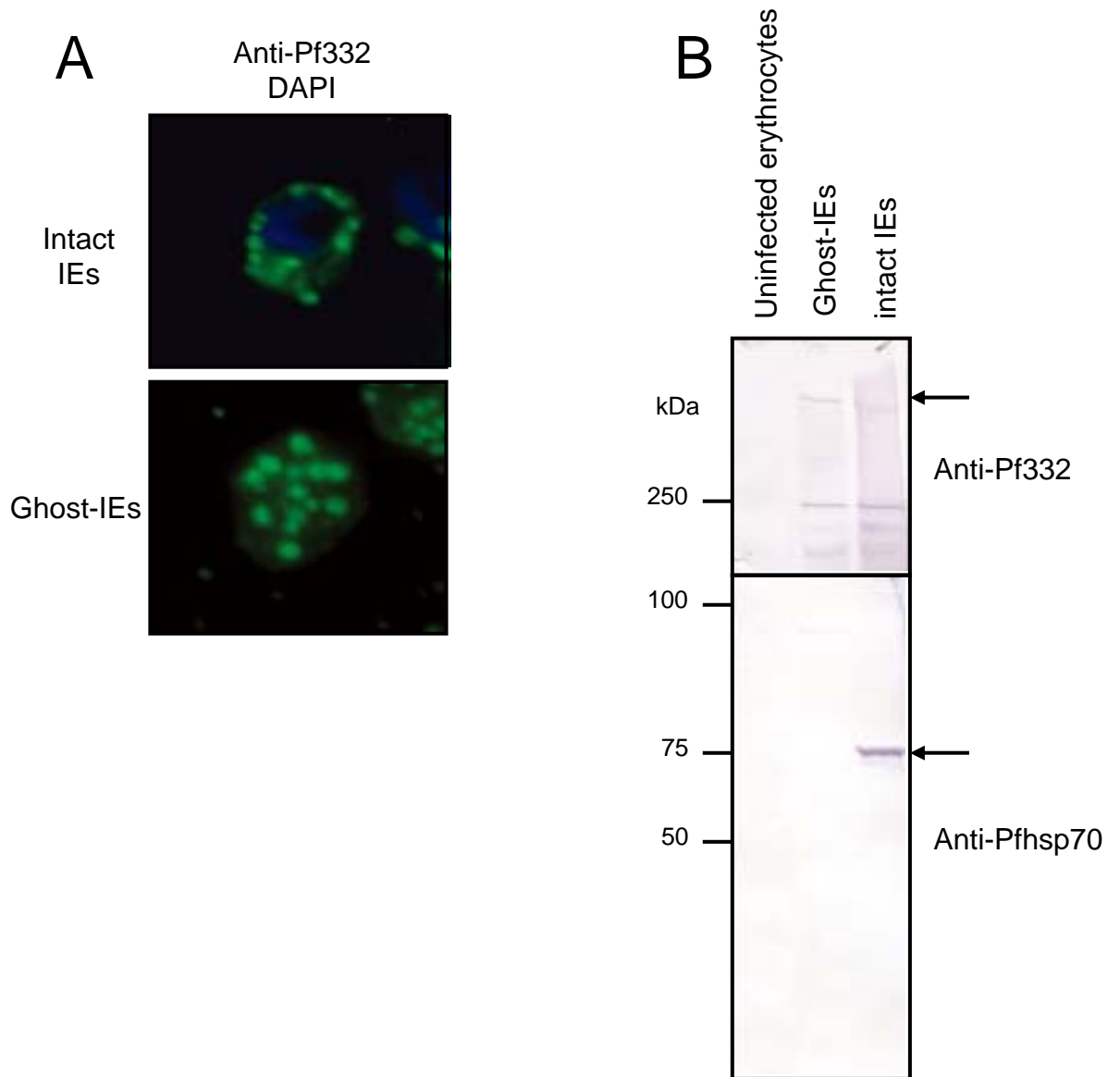

Supplementary -Figure 2

Supplement: Figure S2 — Verification of the purity of ghost fractions. A. Immunofluorescence analysis of the intact IEs and ghost fractions of IEs. Antigen was detected with Pf332 specific antibody. B. Immunoblot analysis of uninfected erythrocytes, ghost fractions and intact IEs. Antigen was detected using specific anti-Pf332 and Pfhsp70 antibodies. (0.02 MB PDF) [file pone.0011747.s002.pdf]
